# Supplementary material for: Resolving the full spectrum of human genome variation using Linked-Reads
Source: Genome Res. 2019 Apr;29(4):635–45. doi: 10.1101/gr.234443.118 (PMC6442396; doi:10.1101/gr.234443.118)
Supplement: Supplemental Material [file supp_gr.234443.118_Supplemental_longranger-2.2.zip › longranger-2.2.tar.bz2!/lr2.2-public/lib/stan/cmdstan/stan/doc/api/html/structstan_1_1gm_1_1whitespace__grammar.html]

Stan: stan::gm::whitespace\_grammar< Iterator > Struct Template Reference


|  |  |
| --- | --- |
|  | Stan  2.5.0  probability, sampling & optimization |


- Main Page
- Related Pages
- Namespaces
- Classes
- Files

- Class List
- Class Index
- Class Hierarchy
- Class Members

All Classes Namespaces Files Functions Variables Typedefs Enumerations Enumerator Friends Macros Pages

- stan
- gm
- whitespace\_grammar

Public Member Functions |
Public Attributes |
List of all members

stan::gm::whitespace\_grammar< Iterator > Struct Template Reference

`#include <whitespace_grammar.hpp>`

Inheritance diagram for stan::gm::whitespace\_grammar< Iterator >:

|  |  |
| --- | --- |
| Public Member Functions | |
|  | whitespace\_grammar () |
|  | |

|  |  |
| --- | --- |
| Public Attributes | |
| boost::spirit::qi::rule< Iterator > | whitespace |
|  | |

## Detailed Description

### template<typename Iterator> struct stan::gm::whitespace\_grammar< Iterator >

Definition at line 11 of file whitespace\_grammar.hpp.

## Constructor & Destructor Documentation

template<typename Iterator >

|  |  |  |  |  |
| --- | --- | --- | --- | --- |
| stan::gm::whitespace\_grammar< Iterator >::whitespace\_grammar | ( |  | ) |  |

Definition at line 13 of file whitespace\_grammar\_def.hpp.

## Member Data Documentation

template<typename Iterator>

boost::spirit::qi::rule<Iterator> stan::gm::whitespace\_grammar< Iterator >::whitespace

Definition at line 16 of file whitespace\_grammar.hpp.

---

The documentation for this struct was generated from the following files:

- src/stan/gm/grammars/whitespace\_grammar.hpp
- src/stan/gm/grammars/whitespace\_grammar\_def.hpp


---

[ Stan Home Page ]

*© 2011–2014,
Stan Development Team.*
